# Predictors of household direct cost of burn injury in adult patients at a tertiary healthcare facility in Ghana: an analytical cross-sectional study in Korle-Bu Teaching Hospital

**DOI:** 10.11604/pamj.2024.48.9.38266

**Published:** 2024-05-07

**Authors:** George Aryee, Veronica Mamle Aborbi, Raymond Essuman, Janet Pereko, Robert Djagbletey, Ebenezer Owusu Darkwa

**Affiliations:** 1 Department of Anaesthesia, University of Ghana Medical School, Korle-Bu, Accra, Ghana,; 2 Reconstructive Plastic Surgery and Burns Centre, Korle-Bu Teaching Hospital, Accra, Ghana

**Keywords:** Burns, costs, treatment, injuries, household

## Abstract

**Introduction:**

treatment of severe burn injury generally requires enormous human and material resources including specialized intensive care, staged surgery, and continued restoration. This contributes to the enormous burden on patients and their families. The cost of burn treatment is influenced by many factors including the demographic and clinical characteristics of the patient. This study aimed to determine the costs of burn care and its associated predictive factors in Korle-Bu Teaching Hospital, Ghana.

**Methods:**

an analytical cross-sectional study was conducted among 65 consenting adult patients on admission at the Burns Centre of the Korle-Bu Teaching Hospital. Demographic and clinical characteristics of patients as well as the direct cost of burns treatment were obtained. Multiple regression analysis was done to determine the predictors of the direct cost of burn care.

**Results:**

a total of sixty-five (65) participants were enrolled in the study with a male-to-female ratio of 1.4: 1 and a mean age of 35.9 ± 14.6 years. Nearly 85% sustained between 10-30% total body surface area burns whilst only 6.2% (4) had burns more than 30% of total body surface area. The mean total cost of burns treatment was GHS 22,333.15 (USD 3,897.58). Surgical treatment, wound dressing and medication charges accounted for 45.6%, 27.5% and 9.8% of the total cost of burn respectively.

**Conclusion:**

the direct costs of burn treatment were substantially high and were predicted by the percentage of total body surface area burn and length of hospital stay.

## Introduction

Burn injuries are a major public health problem causing pain to all sufferers worldwide with higher prevalence in developing regions and among low socioeconomic groups [1]. Globally, burn injuries cause more than 300,000 deaths annually with nearly 95% occurring in low- and middle-income countries [2,3]. Adults have a lower mortality rate of burns compared to children. This may be attributed to the fact that children have thinner skin and may have reduced physiologic reserves. Technical challenges with vascular access, a narrower margin for error in fluid management and the reluctance to subject children to traumatic excisional burn operations may also be contributory factors [4]. The majority of burns (80-90%) occur at home [5]; it is therefore not surprising that in adults, females are at a higher risk of burn-related injuries than males [6]. However, occupational burn injuries are reported to be more likely among young males [7,8]. Most burn-related injuries are caused by thermal energy including scalding and flames, whilst the others are caused by chemical exposure, electricity and ionizing radiation [1].

Treatment of severe burn injury generally requires substantial human and material resources that are related to specialized intensive care, staged surgery and continued rehabilitative and restorative care [9]. This contributes an enormous economic burden to the patients and their families. It has been reported that in the management of injuries in low-to-middle-income countries, burn injuries are associated with the highest economic burden in terms of direct medical costs [10]. Direct medical cost consists of the cost of hospitalization, surgeries, intensive care, nursing and cost of drug and non-drug consumables. Direct non-medical costs cover the cost of maintaining/rehabilitating the burn patient back to his or her normal daily life and include the cost of performance of professional tasks as well as those performed by laypersons/family members [11].

Studies have shown that the cost of burn treatment could be influenced by demographic and clinical factors such as percentage of total body surface area burn (TBSA), gender, length of hospital stay and number of admissions associated with the burns [12,13]. In sub-Saharan African countries such as Ghana, there is a dearth of data on the cost of burn injury as well as the factors that influence the cost of burn care. This study sought to answer the following questions; what are the direct costs of burns care among adult patients, what demographic and clinical factors are associated with direct cost of burn care and if a suitable regression model exists which predicts the direct cost of burn care based on patients´ demographic and clinical characteristics?

## Methods

**Study design:** an analytical cross-sectional study was conducted among adult patients to determine the direct costs of burn care and its associated predictive factors in a large teaching hospital in Ghana.

**Setting:** the study was conducted at the burn centre of the Korle-Bu Teaching Hospital (KBTH) between January and October 2020. The KBTH is the third-largest hospital in Africa and the major national referral centre in Ghana. The KBTH has a bed capacity of 2,000 and sees an average of 29,757 patients monthly. The Burns Centre is one of the centres of excellence of the hospital and under the National Reconstructive Plastic Surgery and Burns Centre. The centre has a 68-bed capacity and sees an average of 128 burn patients per week. The burns unit has a 16-bed capacity intensive care unit (ICU) and a 10-bed capacity high dependence unit (HDU).

**Participants:** the study was conducted among adult burn patients (18 years and above) on admission at the burn centre of the Korle-Bu Teaching Hospital (KBTH).

**Variables:** variables included in the study were grouped into demographic and clinical characteristics, direct medical and direct non-medical costs of burn treatment. Demographic and clinical characteristic variables comprise age, sex, total body surface area (TBSA) burn, cause of burn, degree of burn, duration of hospital stay and type of surgeries performed. Direct medical costs of burn treatment variables consist of the cost of hospital stay, surgical treatment and associated material charges, medication charges, cost of laboratory investigations, cost of transfusion of blood and blood products, cost of meals and charges for special nutrition. Direct non-medical cost variables consist of travel costs and the cost of accommodation for relatives. The outcome of the study was direct costs (sum of direct medical and non-medical costs) of burn treatment whilst the predictors were demographic and clinical characteristics of the participants. Possible confounders such as age were considered in the analysis.

### Data sources and measurement

**Data collection tool:** a data extraction sheet was designed and used to collect data on the demographic and clinical characteristics and direct medical and non-medical costs of burn treatment.

**Data collection:** data were recorded on a data extraction sheet. The age, sex, TBSA burn, cause of burn and degree of burn were recorded. The duration of hospital stay and type of surgeries performed were also noted. Data of the direct medical cost of treatment (cost of hospital stay, surgical treatment and associated material charges, medication charges, cost of wound dressings, cost of laboratory investigations, cost of transfusion of blood and blood products, cost of meals and charges for special nutrition) was obtained from the accounts office of the Burns Centre of KBTH. Components of direct medical costs such as drugs and products purchased outside the centre were also captured by obtaining receipts of such expenditures from the patient or their relatives.

Data on direct non-medical costs such as travel costs and cost of accommodation for relatives who accompanied the patient to the hospital was also recorded. Total costs computed were converted to US dollars at the prevailing mean exchange rate (GHS 5.73 = 1 USD) during the study period of 31^st^ July 2020.

**Sample size:** in a similar study by Gallaher *et al*. [14], the mean daily direct cost of burns was found to be $24.26 ± $6.44 (GHS 139.01 ± GHS 36.06). Using the formula by Rumsey *et al*. [15], a standard deviation of $6.44 (GHS36.06), a confidence level of 95% and a margin of error of 10 units, the sample size of 65 was determined.

**Sampling strategy:** during the study period, folders of patients who satisfied the inclusion criteria were selected at the end of every week and arranged according to the dates of admission. Serial numbers were assigned to the folders and a systematic sampling technique was used to select six (6) patients every week at a sampling interval (K), where K = number of admitted patients for the week (N/6). The first patient was randomly recruited between 1 and K and subsequently every K^th^ patient was recruited. This continued until the required sample size was obtained.

**Data analysis:** data was captured into Microsoft Excel 2016 and secured with a password. At the end of the study period, the data was cleaned and checked for consistency and imported to IBM® SPSS (version 26) for analysis. Missing values were handled by deleting rows with one or more missing values. Quantitative variables such as age, %TBSA burn and direct costs were summarised as mean whereas length of stay was summarised as median (IQR). The degree of burn, cause of burn and type of surgery were summarised as number and proportion. Univariate analysis of variance (ANOVA)/t-test was used to compare the means of the cost of treatment between different categorical variables (sex age groups, percentage TBSA burn, degree of burn, cause of burn and type of surgery). All significant variables associated with direct costs from the univariate analysis were considered for further analysis. A stepwise multiple linear regression was used to determine the best independent predictors of the direct cost of burns. The best model obtained was subject to diagnostics to determine its fitness and accuracy for future prediction of costs by satisfying the key assumptions underlying multiple linear regressions (including lack of multi-collinearity of the independent variables, test of normality and homogenous variance in the residuals). Multi-collinearity was examined using the variance inflation factor (VIF) whilst a normal probability plot was used to check normality and scatterplot as well as the Glejser test for homogenous variance. VIF less than ten (10) indicates the absence of multi-collinearity between the predictors. All p-values ≤0.05 were considered statistically significant.

**Ethical considerations:** the study was approved by the Institutional Review Board of the Korle-Bu Teaching Hospital (Protocol ID: KBTH-IRB/00032/2020). Informed consent was obtained from the participants before enrolment into the study.

## Results

**Demographic characteristics:** a total of sixty-five (65) participants consented and were enrolled in the study with a male-to-female ratio of 1.4: 1 and a mean age of 35.9 ± 14.6 years (range 18 to 92 years). The majority (72.3%) were between the ages of 18 and 39 years whereas 9.2% were 60 years and older.

**Clinical characteristics:** the majority (58.5%) reported to the facility with first-degree burns whilst 7.7% presented with a third-degree burn. The mean (±SE) percentage TBSA burn was 17.2 (±7.4) and ranged between 4.0% and 35%. Nearly 85% sustained between 10-30% TBSA burns whilst 6.2% (4) had burned more than 30% TBSA. About 38.5% had scald burns, 36.9% had flame burns, and 9.2% had electrical burns. For those who had surgery, the majority (90.5%) had split skin grafting (Table 1). The median (IQR) length of hospital stay among participants was 15 (9) days.

**Table 1:** demographic and clinical characteristics of study participants (N=65) enrolled from the burn centre of the Korle-Bu Teaching Hospital (Ghana) between January and October 2020

Variable	Category	n (%)
Sex	Male	38(58.5)
	Female	27(41.5)
	
Age	18-39	47(72.3)
	40-59	12(18.5)
	≥60	6(9.2)
%TBSA	<10	10(15.4)
	10-20	36(55.4)
	21-30	15(23.1)
	>30	4(6.2)
Degree of burn	First degree	38(58.5)
	Second degree	22(33.8)
	Third-degree	5(7.7)
Cause of burn	Fire	24(36.9)
	Electrical	6(9.2)
	Scalds	25(38.5)
	Chemicals	10(15.4)
Type of surgery	Split skin graft	38(90.5)
	Flap cover	2(4.8)
	Escharotomy	1(2.4)
	Debridement+ split skin graft	1(2.4)

TBSA: total body surface area burn

**Direct cost of burn care:** Table 2 shows the mean total direct household cost of the burn was GHS 22,333.15 (USD 3,897.58) with the mean cost of direct medical and direct non-medical cost of burn treatment to be GHS 21,061.23 (USD 3,675.61) and GHS 1,271.92 (USD 221.98) respectively. Surgical treatment, wound dressing and medicine and injection charges accounted for 45.6%, 27.5% and 9.8% of the total cost of burn respectively.

**Table 2:** mean direct household costs of burns (in GHS and USD) and their percentage cost profile of study participants (N=65) enrolled from the burns centre of the Korle-Bu Teaching Hospital (Ghana) between January and October 2020

Cost components	Mean cost		Cost profile (%)
	GHS	(USD)*	
Direct medical costs			
Surgical treatment & associated materials	11,934.00	2,082.72	45.6
Cost of bed occupied	675.00	117.80	0.2
Wound dressings	4,653.28	812.09	27.5
Blood and blood product	246.90	43.09	0.9
Lab tests and examinations	476.18	83.10	2.8
Medication charges	1,664.64	290.51	9.8
Physiotherapy	288.73	50.26	1.4
Meals and beverage charges	863.17	150.64	5.1
Special nutrition charges	259.33	45.26	0.4
Sub-total	21,061.23	3,675.61	93.7
Direct non-medical costs			
Travel cost	255.82	44.65	1.7
Accommodation for relative	403.77	70.47	1.6
Other charges	612.33	106.86	3.0
Sub-total	1,271.92	221.98	6.3
Total direct costs	22,333.15	3,897.58	100

* US$ 1.00 equivalent to GHS 5.73 (Bank of Ghana average monthly interbank exchange rate, July 31st, 2020) GHS: Ghanaian Cedi, USD: United State Dollar

**Demographic and clinical factors associated with direct cost of burn care:** mean direct costs associated with burns treatment significantly varied with varying percentage TBSA, degree of burns and length of hospital stay. Mean direct costs associated with burn treatment also differed significantly with the cause of burn and type of surgical management. However, the mean direct cost did not differ with sex or age (Table 3).

**Table 3:** univariate analysis of direct costs of burn care between the demographic and clinical characteristics of study participants (N=65) enrolled from the burns centre of the Korle-Bu Teaching Hospital (Ghana), between January and October 2020

Variable	Category	Mean ± SE	p-value
**Sex**	Male	18,107.14±2,266.40	0.395
	Female	15,213.09± 2,427.97	
Age	18-39	15,586.13± 1,997.98	0.442
	40-59	20,675.91±3,592.27	
	≥60	19,694.29± 5352.65	
%TBSA	<10	5,570.93±1,625.25	<0.0001*
	10-20	11,632.97±1,071.17	
	21-30	30,361.43±3,242.84	
	>30	42,226.80±1,697.71	
LOS (days)	<10	4,764.42±1,747.27	<0.0001*
	10-20	13,519.49±1,142.75	
	>20	35,777.73±2,755.72	
Degree of burns	First degree	12,251.28±1630.98	0.002*
	Second degree	24,418.25 ±3158.04	
	Third-degree	19,214.92±7256.90	
Cause of burns	Fire	17,604.07±2,740.22	0.028*
	Electrical	1,9071.68±4,317.85	
	Scalds	11,890.53±2,243.08	
	Chemicals	26,463.38±4,929.89	
Types of surgery	Split skin graft	23,320.13±1,970.86	<0.0001*
	Others	24,990.76±5,819.17	
	None	4,899.86±978.69	

TBSA: total body surface area burn, LOS: length of stay, *p-value <0.05: statistically significant; SE-standard error

**Figure 1:**
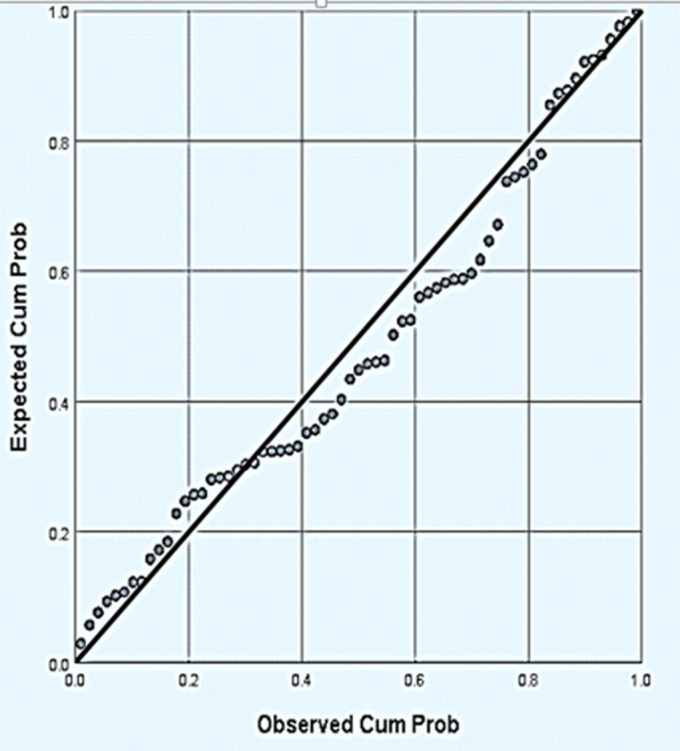
normal probability plot of residuals, the points around the diagonal line represent the model residuals to determine whether the residuals are normally distributed or not

**Figure 2:**
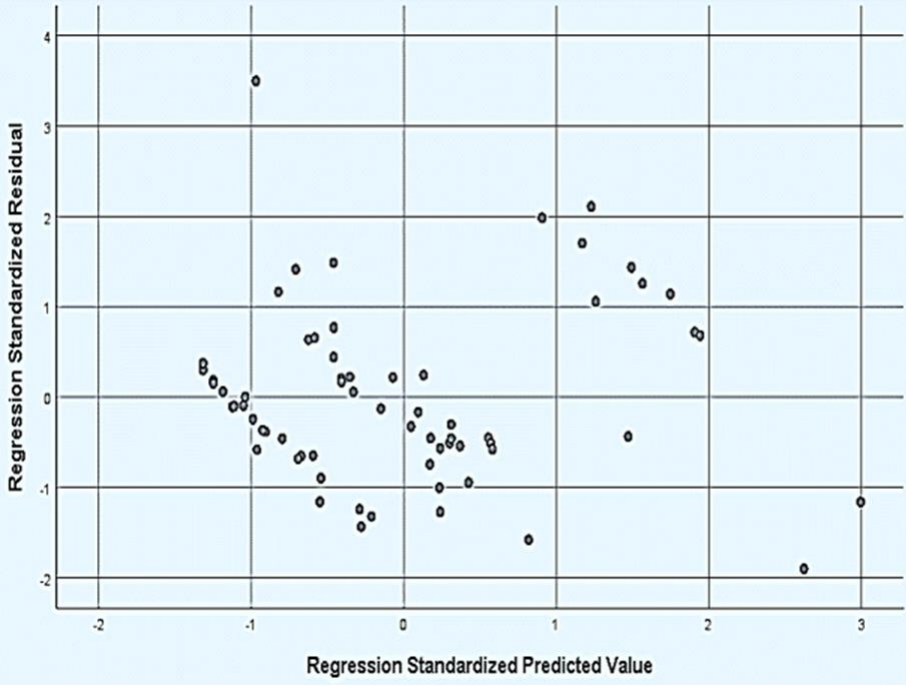
scatterplot of standardized residuals versus standardized predicted value, the points in the plot determine the pattern between the standardized residuals and the standardized predicted values to assess the homogeneity of variance within the residuals

**Regression model predicting factors associated with direct cost of burn care:** a stepwise regression shows % TBSA burn and length of stay (LOS) significantly influenced the direct cost of burn care (p-values <0.0001). Percentage TBSA produced a regression coefficient of 750.87 while that for LOS was 815 with an intercept of -9910.82. A final model which included % TBSA burn and LOS as predictors yielded the model with a coefficient of determination (R^2^) of 85%: direct cost = -9910.82 + (750.87) TBSA + (815) LOS. Model diagnostics yielded the absence of multi-collinearity (VIF_2.78<10_) between the predictors (TBSA and LOS). Figure 1 shows the model residuals (error) terms were normally distributed. The standardized residuals did not indicate any pattern on the scatterplot and further tests conducted showed the residuals had homogenous variance (p-value > 0.05) as shown in Figure 2. Therefore the model fits the data well and is suitable for future prediction of the direct cost of burn care.

## Discussion

Burn injury causes physical and psychological distress and poses a financial burden on affected persons. The cost of burn injury treatment is generally high and influenced by several factors including the patient's demographic and clinical characteristics. This study found surgical procedures and related materials, wound dressing and medication costs contributed substantially to the total direct cost of burn care. TBSA burn and length of hospital stay were found to be independent predictors of the direct cost of burns treatment.

Our study found a predominance of male patients, similar to studies which reported on the epidemiology of burns patients [16-18]. The majority of patients were young adults consistent with the literature [19-21].

We found the direct costs of burns treatment to be high in this study, relative to the low income of majority of Ghanaians. The average annual household expenditure in Ghana is GHS 12,857 (USD 2,243.80) with an annual per capita of GHS 4,574 (USD 798.25) [22], a figure significantly below the average direct cost (GHS 22,3333.15; USD 3,897.58) of burn injury treatment.

The direct cost of burns treatment in this study was markedly lower compared to that reported by studies conducted in developed countries [13,23-25], a finding similar to that by Okafor *et al*. [26]. The variations in the direct costs of burns treatment could be due to differences in the use of technology and treatment protocols. In developed countries, their routine protocol incorporates the use of more advanced technologies such as skin substitutes, ICU care, early excision and grafting for the patients which come with additional costs [23,27].

Even among developing countries, there seems to exist wide differences in the direct cost of burn injury treatment [28]. Okafor *et al*. [26] in their study in Nigeria, a neighbouring West African country, reported a mean direct cost of burn injury treatment to be $7,123.28, nearly twice as that found in the current study. This could be attributed to differences in methodologies or approaches used in the computation of healthcare costs.

Elderly patients are more likely to have underlying comorbidities that could impair wound healing and increase their risk of developing complications. They are thus more likely to have longer hospital stays and higher costs of treatment. In our study, though the cost of care was higher in the elderly (aged ≥60 years), age was not found to significantly impact the cost of care. A finding consistent with that of Latifi *et al*. [12] but contrasting with that of Haikonen *et al*. [24].

Scalds and flame burns were found to be the common causes of burn injury which is consistent with literature [23]. Treatment of chemical burns was the most expensive, followed by flame and scald burns whilst the electrical burns had the lowest direct cost of treatment. This differs from the findings by Anami *et al*. [16] (the highest mean cost was flame burns whilst chemical burns had the least cost) and Sahin *et al*. [29] (mean costs were highest in the electrical burn, followed by flame and scalds). The differences in findings could also be due to differences in patient characteristics and treatment modalities employed.

Similar to other studies, the cost of treatment of burn injury in our study was found to be significantly associated with the extent of burns. [13,24,30]. A multiple linear regression performed in this study found the percentage of TBSA to be an independent predictor of the direct cost of burns consistent with the findings of Matin *et al*. [31] and Kai-Yang *et al*. [32]. Our model revealed that a unit increase in percentage TBSA accounted for a GHS 750.18 (USD 130.92) increase in the mean direct cost of burn injury treatment.

Factors such as the development of complications, the need to manage comorbid conditions, the severity and extent of burns and the need for surgery influence the length of hospital stay. The longer a patient stays in the hospital, the more likely the need for additional medical and surgical interventions and hence the higher the expected cost of treatment. Length of hospital stay has been noted as a significant component of the costs of burn care [27]. Several authors demonstrated that increased length of hospital stay leads to an increased cost of burns treatment [23,28,32]. Similarly, in this study, LOS significantly increased the cost of treatment for burn injury. Our regression model demonstrated that for each day a patient stays in the hospital, the cost of care increased by GHS 815 (USD 142.23).

Studies have asserted that attempts to lessen the length of hospital stay do not automatically translate into considerable cost savings. It has been recommended that efforts to reduce cost have to focus on the modifications and advances in the early treatment and not simply on decreasing the duration of stay [33,34]. Anami *et al*. [16], suggested that healthcare centres in developing countries should put in place approaches to optimise early care and reduce delays in transferring patients to specialized burn centres which would possibly contribute to a decrease in major complications and duration of hospital stay and ultimately reduce the cost of care. Mandal *et al*. [35] also suggested that early excision and grafting can minimise the risk of infection and the administration of antibiotic therapy and reduce the length of hospital stay, in so doing reducing costs.

**Limitations of the study:** this was a single centre thus the findings may not apply to other burn centres. The study did not take into account other costs including indirect and intangible costs which could have significantly influenced the cost of treatment.

## Conclusion

Household direct costs of burn injury treatment were substantially high. Surgical procedures and related materials, wound dressing and medication costs accounted for the most significant proportion of the total direct cost of treating burn injury. The percentage of TBSA and length of hospital stay were independent predictors of the direct cost of burns treatment.

### What is known about this topic

- *Treatment of severe burn injuries contributes to an enormous economic burden on the patients and their families;*
- *The cost of burn treatment is influenced by demographic and clinical factors*.

### What this study adds

- *The management of burn injuries is associated with the high economic burden in terms of direct medical costs in low-to-middle-income countries;*
- *There is a dearth of data on the cost of burn injuries in sub-Saharan Africa;*
- *This study develops a regression model to predict the factors (demographic and clinical factors) associated with the cost of burn injury treatment in sub-Saharan African countries*.
